# Breast Cancer Diagnosis in Coronavirus-Era: Alert From Italy

**DOI:** 10.3389/fonc.2020.00938

**Published:** 2020-05-22

**Authors:** Gianluca Vanni, Marco Pellicciaro, Marco Materazzo, Leonardo Palombi, Oreste Claudio Buonomo

**Affiliations:** ^1^Breast Unit, Department of Surgical Science, Tor Vergata University (PTV), Rome, Italy; ^2^Department of Biomedicine and Prevention, Tor Vergata University, Rome, Italy

**Keywords:** COVID-19, breast cancer, screening, diagnosis, Italy

SARS-COV-2 is currently spreading all over the world, exhibiting a trend that has been defined by the WHO as a pandemic. Italy was the first European country to be involved in this outbreak. Based on the latest data, in our country, 224,760 people have been confirmed positive for SARS-COV-2 with a case fatality rate reaching up to 10% (1). These numbers define what is called a national emergency, which consequentially implies a total reorganization of the Health System. In this regard, most routine preventive activities have been scaled down, including active screening campaigns. Breast Units have been strongly affected by the emergency and its consequential restrictions and constraints. Breast pathology, as is well-known, has a strong impact on women health, survival, and quality of life. In fact, Breast Cancer is the most common neoplasia in females, affecting approximately one out of nine women (2). The breast-diagnostic path may begin in three different ways: screening, follow-up with clinical examination, or emergencies related to a new palpable nodule. Every year, around 53,000 women receive a breast cancer diagnosis in Italy (2). From 2015 to 2018, around 74% of women aged between 50 and 69 underwent preventive screening: 54.6% belonged to organized programs while 19.3% performed it voluntarily (3). This method enabled the diagnosis of around 16% of breast cancer (2). Over the years, the National Health System has invested many resources in order to provide early diagnoses of breast cancer. This has been made possible thanks to the empowerment of screening campaigns and through patients' sensibilization to follow periodical controls. As a consequence, the increase in early diagnoses of breast cancer lead to improved prognoses, more conservative surgical treatments with better aesthetical results, as well as reduced health system costs (4). Long-term screening resulted in a reduction of up to 30% in 8 years among the incidence of local advanced breast cancer rate (5). In the pre-screening era, more than 50% of breast cancer presented with a primary tumor sized more than 2 cm and/or an associated axillary lymphadenopathy (6). Around 44% of women, during the screening program, receive an early diagnosis for breast cancer, and this, together with the evolution and progress of treatments, has resulted in a drastic reduction in patient mortality, with an overall survival rate of 87% at 5 years (2, 7).

In view of the above, during this healthcare emergency, surgical ambulatory activities as well as radiological exams are currently granted only for emergencies; screening and elective activities are suspended, shifting all the resources to be made available to the essential services. Cessation of elective activities, screening programs, and the drastic reduction of services restrict the evaluation to only clinical observation of palpable lesions.

What will happen to patients that cannot undergo screening or follow-up due to the outbreak? How many patients with advanced breast neoplasia will be diagnosed at the end of the emergency? How many patients will require the use of neoadjuvant therapy, secondary to the local advanced diagnosis of cancer presentation?

Based on these considerations and reflecting these questions, we hope that the national and international scientific community will be able to design some control and management programs.

The uncertainty regarding the outbreak's ending time and the gradual reversion to a normal situation has brought attention to a need for some new protocols in order to manage patients who were affected by the profound changes of this period. If only a single patient who routinely undergoes screening misses a single mammography, the consequences are limited; if we miss hundreds of thousands of mammograms, however, the impact is extremely severe in terms of lost lives, costs, and the burden on the healthcare system.

Unfortunately, tumor doubling times are not constant. Such studies estimating the mean of tumor doubling times varied from 45 to 260 days, and this very inaccurate measuring is unhelpful in determining the effect of screening delays on breast cancer survival (8). It is safe to estimate that, in 6 months, up to 50% of cases of breast cancer could exhibit a growth of more than the size of a centimeter (7, 8).

In our opinion, in the coming future we will assist in a shift toward a clinical presentation of more advanced breast cancer. This could impair the oncological outcome, worsen the quality of life due to more invasive surgery, chemotherapy, and radiotherapy, and also increase the relative cost for the Public Health System (4).

In order to organize our departments and hospitals as efficient as possible and to be ready to restart, epidemiological studies could be useful tools to evaluate the impact of screening programs in order to prevent a setback to where we were 20 years ago when a 2 cm lesion was considered an early diagnosis of BC (5).

## Author Contributions

GV, MP, MM, LP, and OB contributed equally to this work, including the conception, design, drafting the work, and final approval of the version to be published.

## Conflict of Interest

The authors declare that the research was conducted in the absence of any commercial or financial relationships that could be construed as a potential conflict of interest.
